# Indium-contacted van der Waals gap tunneling spectroscopy for van der Waals layered materials

**DOI:** 10.1038/s41598-021-97110-z

**Published:** 2021-09-07

**Authors:** Dong-Hwan Choi, Kyung-Ah Min, Suklyun Hong, Bum-Kyu Kim, Myung-Ho Bae, Ju-Jin Kim

**Affiliations:** 1 Department of Physics, Jeonbuk National University, Jeonju, 54896 Republic of Korea; 2 Korea Research Institute of Standards and Science, Daejeon, 34113 Republic of Korea; 3 Department of Physics and Graphene Research Institute, Sejong University, Seoul, 05006 Korea; 4 Department of Nano Science, University of Science and Technology, Daejeon, 34113 Republic of Korea

**Keywords:** Electronic properties and materials, Electronic and spintronic devices

## Abstract

The electrical phase transition in van der Waals (vdW) layered materials such as transition-metal dichalcogenides and Bi_2_Sr_2_CaCu_2_O_8+*x*_ (Bi-2212) high-temperature superconductor has been explored using various techniques, including scanning tunneling and photoemission spectroscopies, and measurements of electrical resistance as a function of temperature. In this study, we develop one useful method to elucidate the electrical phases in vdW layered materials: indium (In)-contacted vdW tunneling spectroscopy for 1*T*-TaS_2_, Bi-2212 and 2*H*-MoS_2_. We utilized the vdW gap formed at an In/vdW material interface as a tunnel barrier for tunneling spectroscopy. For strongly correlated electron systems such as 1*T*-TaS_2_ and Bi-2212, pronounced gap features corresponding to the Mott and superconducting gaps were respectively observed at *T* = 4 K. We observed a gate dependence of the amplitude of the superconducting gap, which has potential applications in a gate-tunable superconducting device with a SiO_2_/Si substrate. For In/10 nm-thick 2*H*-MoS_2_ devices, differential conductance shoulders at bias voltages of approximately ± 0.45 V were observed, which were attributed to the semiconducting gap. These results show that In-contacted vdW gap tunneling spectroscopy in a fashion of field-effect transistor provides feasible and reliable ways to investigate electronic structures of vdW materials.

## Introduction

Van der Waals (vdW) layered materials such as two-dimensional (2D) transition-metal dichalcogenides (TMDCs) and Bi_2_Sr_2_CaCu_2_O_8+*x*_ (Bi-2212) have shown various electronic phases that emerge from many-body features such as a charge density wave (CDW) or superconductivity, depending on the temperature and carrier density^1–9^. The vdW interface between dissimilar vdW materials have allowed to investigate the electronic structures of such strongly correlated electron systems. For instance, transport spectroscopy in TMDC/Bi-2212 vdW junctions has revealed gap natures due to the many-body features via the formation of a metal/superconductor proximity junction with a low vdW contact resistance^10,11^. For tunneling spectroscopy, on the other hand, a graphite/Bi-2212 interface provided a vdW gap tunnel junction, enabling tunneling spectroscopy for the Bi-2212 superconductor^11,12^. However, it could be hard to apply the graphite to TMDCs for vdW tunneling spectroscopy because graphene/TMDC contacts have been used to form an Ohmic contact^13^. Thus, for feasible and reliable tunneling spectroscopy for vdW materials, it is crucial to seek a material to form a vdW tunneling gap with any vdW materials. With this purpose, we focus on indium (In) metal in this study.

For evaporated-metal/TMDC contacts, only indium (In) metal has shown vdW contact for TMDCs^14,15^, owing to its low evaporation temperature of ~ 500 °C. In this case, the In vdW contact provides an Ohmic contact for a few-layer TMDCs. However, the vdW contact at a cryogenic temperature could provide a vacuum tunneling gap with a high contact resistance that makes the flow of current sensitive to the electronic DOS at the interface. Indeed, vdW gap tunneling spectroscopy based on a field-effect transistor (FET) design with carbon nanotubes (CNTs) with In metal contacts was demonstrated, recently^16,17^. In this previous work, the local conductance peaks observed in the conductance vs bias voltage plot were shown to originate from the van Hove singularities corresponding to the sub-band structures of semiconducting and metallic CNTs.

In the present study, we apply a type of FET with In contacts for various vdW layered materials (i.e., 1*T*-TaS_2_, Bi-2212, and 2*H*-MoS_2_) to demonstrate that In-contacted vdW gap tunneling spectroscopy is a feasible method to investigate the electrical DOS of vdW layered materials. For the experiments with 1*T*-TaS_2_, the zero-bias resistivity showed a sudden increase at *T* ~ 180 K as the temperature was lowered. At *T* = 4 K, a plot of the differential conductance for various bias voltages revealed the emergence of an energy gap, i.e., the Mott gap edge, which has been observed in the same material only using scanning tunneling and photoemission spectroscopies^18–21^. The In/Bi-2212 junction also showed a superconducting gap of ~ 58 meV at *T* = 4 K. The gap feature slightly decreased with increasing gate voltage, which indicates that the high-temperature superconductivity could be controlled by electric fields^12^. Finally, for ~ 10 nm-thick MoS_2_ FETs, we observed gap features at energy levels of ~ 0.9 eV in differential conductance vs bias voltage curves recorded at *T* = 4 K, corresponding to a semiconducting bandgap. The formation of the tunneling barrier with a high contact resistance for ~ 10 nm-thick MoS_2_ is inconsistent with the Ohmic contacts for few-layer (thickness ⪅ 4 nm) MoS_2_ flakes with In contacts, which might be related to the location of the Fermi level of an In electrode with respect to the bandgap, depending on the thickness of the MoS_2_ layer. The naturally formed vdW tunnel gap without any artificial insulating barrier is very robust under varying temperature. Our work provides simple and reliable identification of electronic DOS using the simple FET geometry for the vdW materials without sophisticated tools such as scanning tunneling microscope.

## Measurements and results

### Experiments

**Figure 1:**
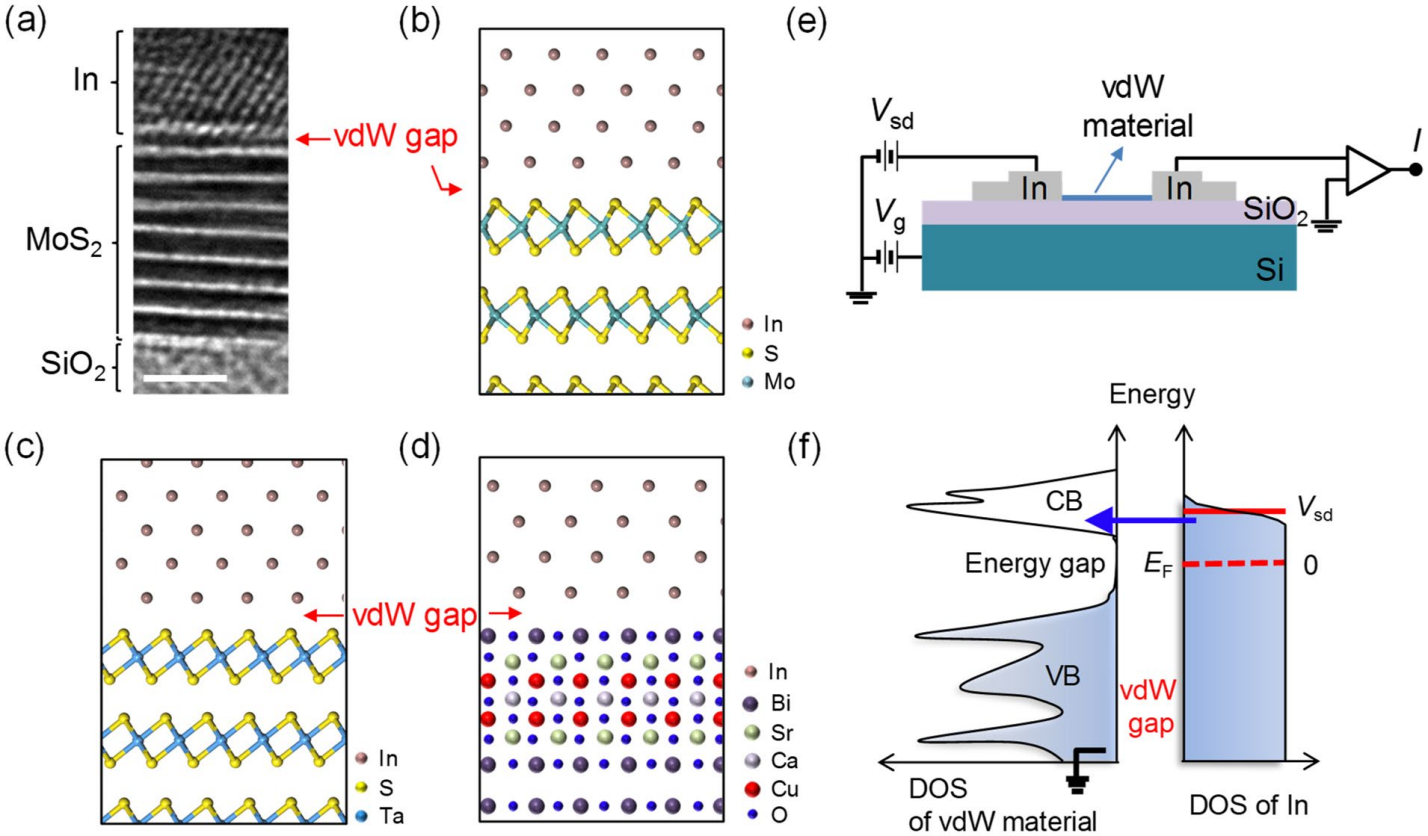
In-contacted vdW gap tunneling spectroscopy for vdW layered materials. (**a**) Cross-sectional TEM image of an In/few-layered MoS_2_ junction. Scale bar: 2 nm. (**b**)–(**d**) Atomic structures at In/2*H*-MoS_2_, In/1*T*-TaS_2_, and In/Bi-2212 interfaces (schematic), respectively. Here, Atomic structures of In/2*H*-MoS_2_ and In/1*T*-TaS_2_ were prepared by the DFT calculations. (**e**) Schematic of the measurement configuration of a device with an FET design. (**f**) Schematic of the electronic DOS at a vdW material/In interface; this schematic describes the tunneling spectroscopy. CB and VB are the conduction and valence bands of MoS_2_, respectively. *E*_F_ is the Fermi energy of the In electrode. Blue and white regions indicate states occupied and unoccupied by electrons, respectively. Under a zero-bias condition, the *E*_F_ is located at the midgap in the energy-gap region of the vdW material (dashed red line). Under a bias voltage of *V*_sd_, the Fermi level of In becomes aligned with the empty states of the CB, resulting in a tunnel current through the vdW gap. The differential conductance obtained from the tunnel current at a cryogenic temperature reflects the DOS of the vdW material.

Single crystals of 1*T*-TaS_2_ and Bi-2212 were grown by the usual iodine transport method and solid-state-reaction methods, respectively. A 2*H*-MoS_2_ single crystal was commercially purchased (HQ Graphene). We fabricated vdW material-based FETs with In contacts for TaS_2_, Bi-2212, and MoS_2_, by carrying out several microfabrication processes. The vdW material flakes on a 500 nm-thick SiO_2_/Si substrate were prepared via mechanical exfoliation from the vdW materials. We deposited 100 nm-thick In electrodes onto a multilayer flake using traditional electron-beam lithography and thermal deposition processes^16^. To investigate the quality of the In/vdW material, we collected cross-sectional transmission electron microscopy (TEM) image of an In/few-layered MoS_2_ junction (Fig. 1a), where the thermally deposited In did not show invasion into the MoS_2_ layer, resulting in a well-defined vdW gap. Atomic structures of interfaces between In and two vdW materials of 2*H*-MoS_2_ and 1*T*-TaS_2_ with vdW gaps were prepared by the density functional theory (DFT) calculations as shown in Fig. 1b,c, respectively. The schematic atomic structure for In/Bi-2212 is also plotted in Fig. 1d. Figure 1e shows a schematic of a completed device for the vdW tunneling spectroscopy experiment (Fig. 1f), where the highly doped Si substrate serves as a back-gate electrode. For basic electrical characterizations of the three vdW materials such as carrier density (*n*_H_) and Hall mobility (*μ*_H_), we performed independent electrical measurements for 1*T*-TaS_2_ and 2*H*-MoS_2_. We measured the resistivity of 1*T*-TaS_2_ as a function of *T* in a four-probe configuration (see Supplementary Fig. 1), where *ρ* ~ 0.1 Ω cm for 50 < *T* < 220 K. This is a similar *ρ* range with a previous report^22^, thus we expect that *n*_H_ and *μ*_H_ show a similar trend with temperatures. For 2*H*-MoS_2_, we performed the Hall measurement with varying *T* and back-gate voltage (see Supplementary Fig. 2). For Bi-2212, we estimated *n*_H_ and *μ*_H_ from literatures showing a similar *T*_c_ with a similar hole doping^23,24^. The list of *n*_H_ and *μ*_H_ for the three vdW materials was shown in Table 1.

**Table 1:** Representative carrier type, carrier density (*n*_H_) and Hall mobility (*μ*_H_) for three vdW materials.

	1*T*-TaS_2_^22^	Bi-2212^23,24^	2*H*-MoS_2_ (thickness: 6 nm)
Carrier type and *n*_H_	For 4 < *T* < 200 K Hole (0.4–5) × 10^19^ cm^−3^	For *T*_c_ < *T* < 300 K Hole (3–6) × 10^21^ cm^−3^	For 2 < *T* < 300 K Electron (1–2) × 10^13^ cm^−2^
	For 200 < *T* < 300 K Electron (0.3–1) × 10^22^ cm^−3^		
*μ*_H_ (cm^2^ V^−1^ S^−1^)	For 1 < *T* < 10 K 1–10	For 3.2–7 nm thicknesses 3–11	For 2 < *T* < 300 K 3000–20
	For 70 < *T* < 300 K 30–0.3		

### vdW gap tunneling spectroscopy for 1*T*-TaS_2_

**Figure 2:**
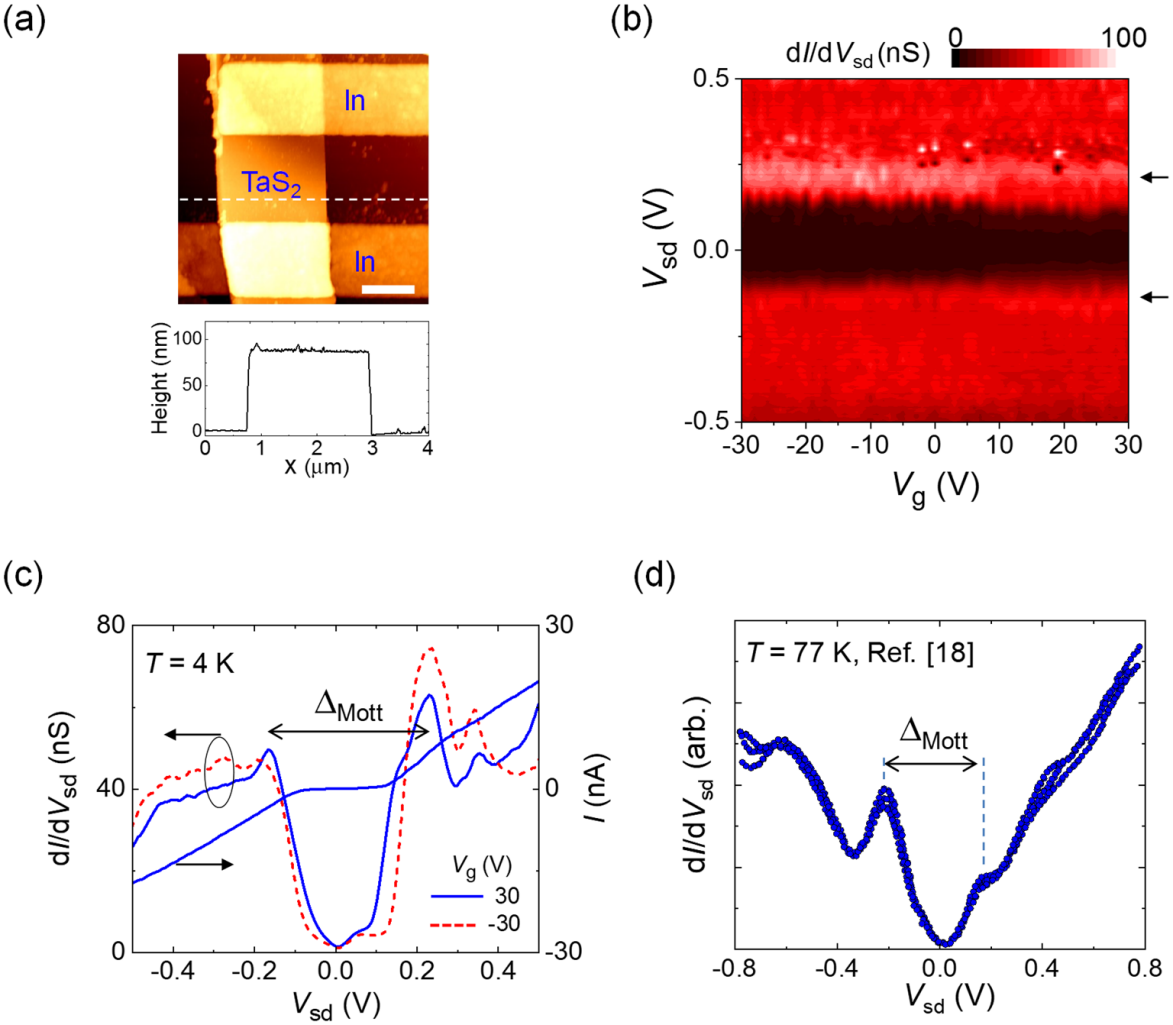
vdW gap tunneling spectroscopy for 1*T*-TaS_2_. (**a**) Upper panel: AFM image of a 1*T*-TaS_2_ FET. Scale bar: 1 µm. Lower panel: height profile along the dashed white line in the upper panel. (**b**) d*I*/d*V*_sd_ as a function of *V*_sd_ and *V*_g_ at *T* = 4 K. The two arrows indicate the conductance shoulders corresponding to a gap feature. (**c**) d*I*/d*V*_sd_–*V*_sd_ curves at *V*_g_ =  ± 30 V and *I*–*V*_sd_ curve at *V*_g_ = 30 V. These curves clearly show an energy-gap feature, indicated by the Mott gap (*Δ*_Mott_) with a magnitude of ~ 0.4 eV. (**d**) Conventional scanning tunneling spectra obtained from the same 1*T*-TaS_2_ single crystal at *T* = 77 K, which shows a similar *Δ*_Mott_ size with that in (**c**). (**d**) is reproduced with permission from^18^, 1994 AMERICAN PHYSICAL SOCIETY.

The upper panel of Fig. 2a shows an atomic force microscopy (AFM) image of a fabricated TaS_2_ device, where the channel length (*L*) and width (*W*) are 1.7 μm and 2.2 μm, respectively. The lower panel of Fig. 2a shows the height profile along the dashed white line in the upper panel, indicating that the thickness of TaS_2_ was ~ 88 nm. Figure 2b shows d*I*/d*V*_sd_ as a function of the source–drain voltage (*V*_sd_) and back-gate voltage (*V*_g_) at *T* = 4 K, which shows a substantial suppression of conductance near zero bias, along with conductance shoulders (indicated by two arrows). In the *I*–*V*_sd_ curve corresponding to *V*_g_ = 30 V in Fig. 2c, a relatively flat current region is observed near zero bias, which corresponds to the conductance dip region at the same *V*_g_ value in Fig. 2b. The d*I*/d*V*_sd_–*V*_sd_ curve corresponding to *V*_g_ = 30 V shows a clear gap feature indicated by the bidirectional arrow, which was also observed at *V*_g_ =  − 30 V in Fig. 2c. We speculated that the observed gap is related to the Mott gap (*Δ*_Mott_), exhibiting a gap size of ~ 0.4 eV. The Mott transition in multilayer 1*T*-TaS_2_ has been previously shown to be developed in the temperature range 180 ≤ *T* ≤ 210 K, accompanied by a transition from the nearly-commensurate CDW (NCCDW) phase to the commensurate CDW (CCDW) phase, as revealed by scanning tunneling spectroscopy and photoelectron spectroscopy^18,19^. A recent study based on resistance measurements as a function of *T* conducted by using a gate-controlled Li^+^-ion intercalation method showed that the CCDW phase changed to the NCCDW phase with increasing gate voltage at *T* = 10 K^2^. In our case, the width of the conductance dip was nearly constant for *V*_g_ values spanning 60 V, possibly because of relatively less change in the carrier density in our gating method with a 500 nm-thick dielectric SiO_2_ layer. In addition, in Fig. 2d, we displayed already reported d*I*/d*V*_sd_–*V*_sd_ curves obtained by conventional scanning tunneling spectroscopy with the same crystal used in this study^18^. Both of them showed a similar *Δ*_Mott_ size of ~ 0.4 eV, thus we believe that In-contacted vdW gap tunneling spectroscopy provides a credible method to study the electronic states in vdW materials.

**Figure 3:**
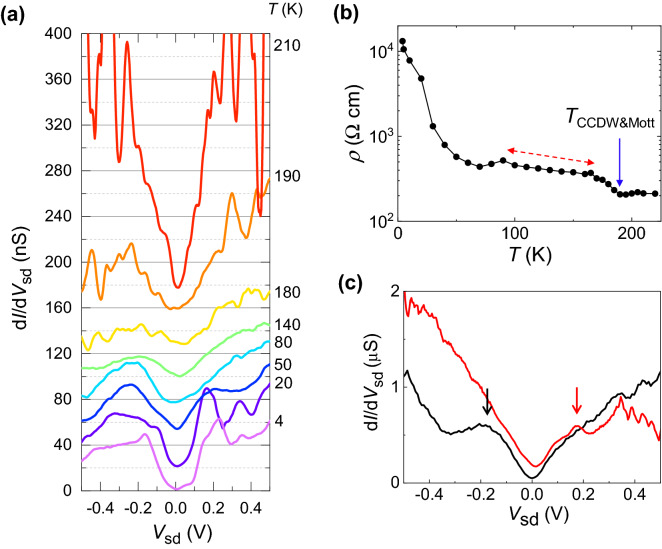
Temperature dependence of Mott gap. (**a**) d*I*/d*V*_sd_ as a function of *V*_sd_ at *V*_g_ = 30 V for various temperatures, where each curve is vertically shifted for clarity. (**b**) Zero-bias resistivity at *V*_sd_ = 0 V as a function of *T*, as obtained from (**a**). Near *T* ~ 180 K, the resistivity suddenly increased with decreasing *T*, as indicated by the arrow. The region indicated by a dashed bidirectional arrow indicates a pseudogap region. (**c**) d*I*/d*V*_sd_ as a function of *V*_sd_ for two other 1*T*-TaS_2_ devices, each of which showed a gap feature, as indicated by the two arrows.

To investigate the phase transition, we obtained d*I*/d*V*_sd_–*V*_sd_ curves at *V*_g_ = 30 V over the temperature range 4 ≤ *T* ≤ 210 K, as shown in Fig. 3a, where the curves are vertically shifted as much as 20 nS for clarity. The pronounced gap feature at *T* = 4.2 K was smeared with increasing *T* up to *T* ~ 140 K and became featureless at *T* ≥ 180 K, whereas the conductance dip near zero bias was still observed. Figure 3b shows the zero-bias resistivity as a function of *T*, as extracted from Fig. 3a at *V*_sd_ = 0 V. A sudden increase is observed at *T* ~ 180 K (indicated by an arrow) with decreasing *T*. This behavior was found to be consistent with previous observations of the Mott transition accompanied by the phase transition from the NCCDW phase to the CCDW phase near this temperature, *T*_CCDW&Mott_^19,20^. In that Mott transition, a gap was not fully opened in the investigated *T* region, resulting in a so-called a pseudogap structure^19,20^. In our case, such behavior was observed in the region indicated by a dashed bidirectional arrow in Fig. 3b, where the zero-bias resistivity monotonically increases with decreasing *T*. The d*I*/d*V*_sd_–*V*_sd_ curves corresponding to 80 ≤ *T* ≤ 180 K in Fig. 3a show pseudogap-hump structures at *V*_sd_ ~  ± 0.3 V with a finite zero-bias conductance, representing a small but finite density of state at the Fermi energy (*E*_F_). Importantly, at *T* < 60 K, the resistivity increases substantially faster with decreasing *T* (see Fig. 3b) and the zero-bias conductance finally decreases to nearly zero at *T* < 20 K (Fig. 3a), where conductance peaks corresponding to the gap edges are clearly observed at *V*_sd_ ~  ± 0.2 V, as shown in Fig. 3a (see also Fig. 2c). This result indicates that the Mott gap was fully developed at *T* < 20 K^21^. To test the reproducibility of these results, we also fabricated two additional 1*T*-TaS_2_ devices with a thickness similar to that of the first 1*T*-TaS_2_ device, and the curves for each device showed a clear conductance peak at one polar *V*_sd_ of − 0.20 V and 0.18 V at *T* = 4 K, respectively, as shown in Fig. 3c (vertical arrows). Asymmetric gap features have been frequently observed in tunneling spectroscopy results for two electrodes (tip and metal contacts) with substantially different contact resistance levels (see Fig. 2d)^18^.

### vdW gap tunneling spectroscopy for Bi-2212

**Figure 4:**
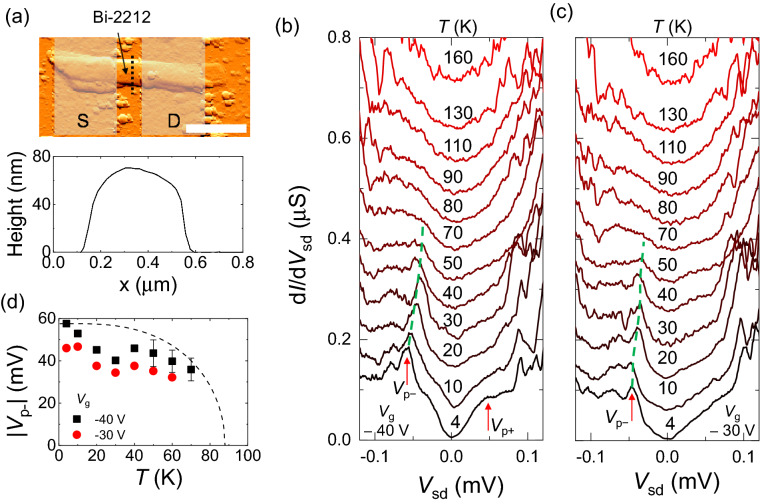
vdW gap tunneling spectroscopy for Bi-2212. (**a**) Upper panel: AFM image of a Bi-2212 device. Scale bar: 1 μm. Lower panel: height profile along the dashed black line in the upper panel. (**b**), (**c**) d*I*/d*V*_sd_ as a function of *V*_sd_ for various temperatures, as obtained at *V*_g_ =  − 40 and − 30 V, respectively. Dashed green lines follow the gap edges as *T* is varied. (**d**) Closed squares and circles: |*V*_p−_| as a function of *T* for *V*_g_ =  − 40 and − 30 V, respectively. Dashed curve: a plot based on Eq. (1) for comparison.

The as-grown Bi-2212 crystal used in the present study exhibits a slightly hole-overdoped character; the superconducting transition temperature (*T*_c_) measured from other crystals obtained from the same batch was ~ 88 K^25^. The upper panel of Fig. 4a shows an AFM image of a Bi-2212 device with *L* ~ 0.4 μm and *W* ~ 0.4 μm. The lower panel of Fig. 4a shows a height profile along the dotted line depicted in the upper panel of Fig. 4a, indicating that the thickness of the Bi-2212 is ~ 70 nm. Figure 4b shows d*I*/d*V*_sd_ as a function of *V*_sd_ at *V*_g_ =  − 40 V for various temperatures, where the curves are vertically shifted as much as 50 nS. At *T* = 4 K, the gap feature was observed at *V*_sd_ ~  − 58 and 50 mV, which are assigned as *V*_p−_ and *V*_p+_, respectively, as indicated by two red arrows in the figure. The observed gap sizes are consistent with previous observations of the superconducting gap energy, $\Delta$^26,27^. The peak signature at *V*_p+_ is relatively weak compared with that at *V*_p−_, similar to the TaS_2_ case. The *V*_p−_ value corresponding to the conductance peak decreases with increasing *T*, as indicated by the dashed green line, and is smeared out near the *T*_c_ at *T* = 80 K. Figure 4d shows |*V*_p−_| as a function of *T* at *V*_g_ =  − 40 V, where |*V*_p−_| decreases with decreasing *T*. For comparison, we added a dashed curve representing $\Delta \left( T \right)$/*e* based on the expression^28^

1 $$\Delta \left( T \right) = \Delta_{0} \tanh \left( {\frac{\pi }{a}\sqrt {b\left( {\frac{{T_{{\text{c}}} }}{T} - 1} \right)} } \right)$$

where *e* is the elementary charge; $\Delta_{0}$ = 58 meV, *a* = 2.14, and *b* = 4/3 for a weak-coupling 2D d-wave superconductor; and *T*_c_ = 88 K. Although we lack exact information about the *T*_c_ at *V*_g_ =  − 40 V, the data qualitatively follows Eq. (1). Thus, we conclude that the gap feature originates from the superconducting gap. We also measured d*I*/d*V*_sd_ as a function of *V*_sd_ at *V*_g_ =  − 30 V as *T* was varied (Fig. 4c). As depicted by the dashed green line, the superconducting gap energy decreases with increasing *T*. However, we note that the gap feature relatively weakens at *V*_g_ =  − 30 V compared with that at *V*_g_ =  − 40 V. For instance, at *V*_g_ =  − 30 V, *V*_p__−_  decreases to approximately − 46 mV at *T* = 4 K, accompanied by a reduction of the conductance peak height. The conductance peak corresponding to the superconducting gap edge at the positive *V*_sd_ region (*V*_p+_) even disappears at *T* = 4 K. For comparison, we added |*V*_p−_| as a function of *T* obtained at *V*_g_ =  − 30 V in Fig. 4d. Recently, Liao et al. reported that the superconductor–insulator transition in Bi-2212 could be achieved using a back-gate method with a solid ionic conductor, where the carrier density was modulated by intercalated Li^+^ ions^12^. This result may indicate that the superconductivity of Bi-2212 can also be manipulated by a back-gate field with a SiO_2_/Si substrate design as our case, although further study is needed to quantitatively demonstrate the feasibility of this concept.

### vdW gap tunneling spectroscopy for 2*H*-MoS_2_

**Figure 5:**
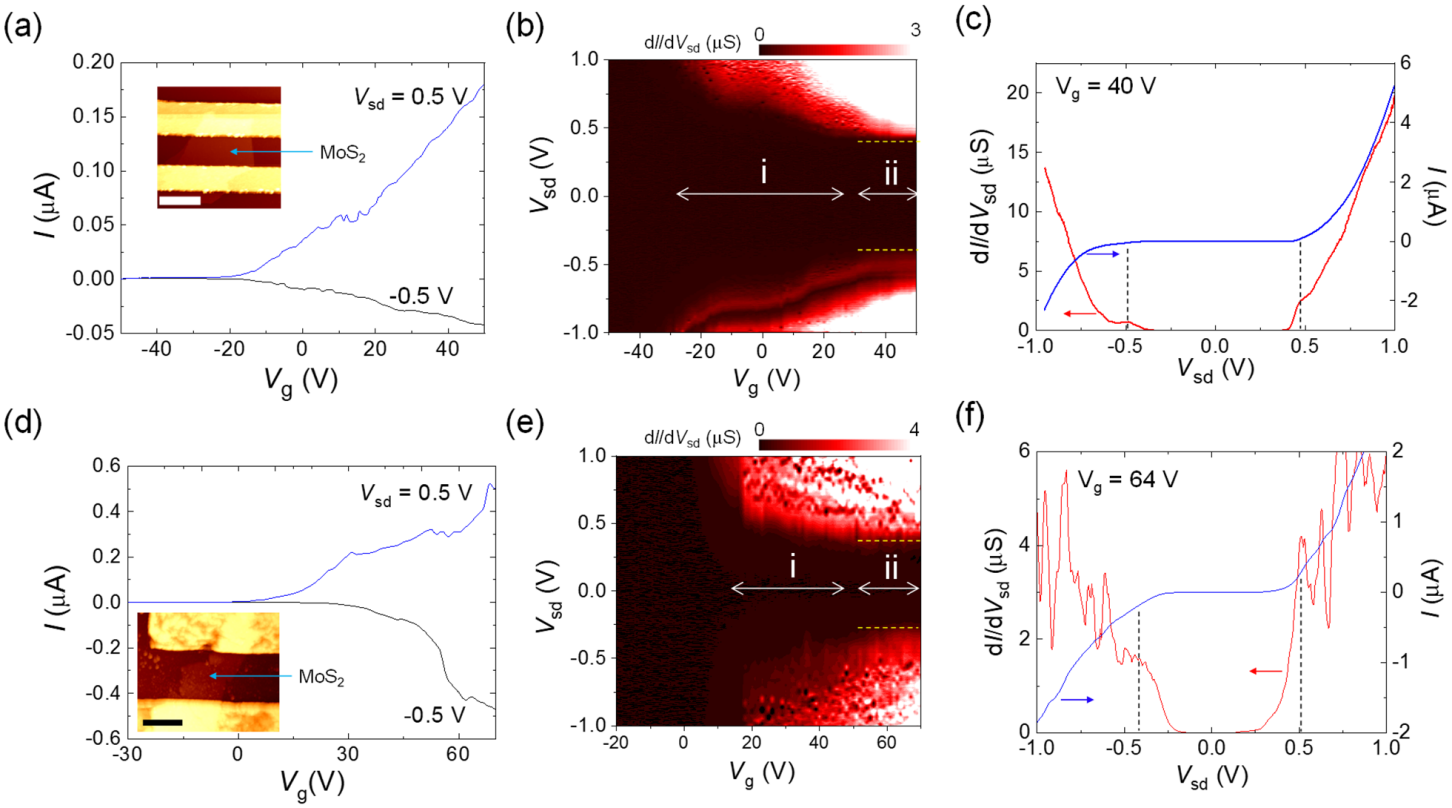
vdW gap tunneling spectroscopy for 2*H*-MoS_2_. (**a**), (**d**) Current vs. back-gate voltage (*I*–*V*_g_) curves at *V*_sd_ =  ± 0.5 V and *T* = 4 K for MS1 and MS2, respectively. Insets of (**a**), (**d**): AFM images of MS1 (scale bar: 1 μm) and MS2 (scale bar: 2 μm), respectively. The thickness of the MoS_2_ flakes in both cases was ~10 nm, corresponding to ~ 14 MoS_2_ layers. (**b**), (**e**) Differential conductance (d*I*/d*V*_sd_) as a function of *V*_sd_ and *V*_g_ of MS1 and MS2, respectively. The zero-conductance region on the *V*_sd_ axis became smaller with increasing *V*_g_ in region “i” and did not change in region “ii”. The two dashed lines indicate the edges of the bandgap. (**c**), (**f**) d*I*/d*V*_sd_ and *I* as a function of *V*_sd_ at *V*_g_ = 40 and 64 V for MS1 and MS2, respectively. The left and right vertical dashed lines located at the conductance shoulders correspond to the edges of the conduction and valence bands, respectively.

The inset of Fig. 5a shows an AFM image of the MoS_2_ FET (MS1) with a thickness of ~ 10 nm. The MS1 device showed a traditional *n*-type transfer curve for *V*_sd_ = ± 0.5 V at *T* = 4 K (Fig. 5a), where the *I*–*V*_g_ curves show an asymmetric behavior depending on the polarity of *V*_sd_. Figure 5b shows a d*I*/d*V*_sd_ map as a function of *V*_sd_ and *V*_g_. In Schottky FETs, the zero-conductance region observed when *V*_sd_ is swept in a depletion state decreases with positively increasing *V*_g_ for an *n*-type device because the width of the Schottky barrier decreases with positively increasing *V*_g_. Although the conductance map in Fig. 5b appears to show such behavior for *V*_g_ < 30 V (region i), a robust zero-conductance region that is independent of the change in *V*_g_ was observed at − 0.45 V ≤ *V*_sd_ ≤ 0.45 V (see dashed yellow lines) in the *V*_g_ region labeled as region ii. Figure 5c shows *I* and d*I*/d*V*_sd_ as functions of *V*_sd_ at *V*_g_ = 40 V in region ii. Conductance shoulders, indicated by the two vertical dashed lines, are separated from each other by an energy scale of ~ 0.96 eV, which is close to the interval bandgap of ~ 1.2 eV expected for multilayer MoS_2_^29^.

To confirm the consistency of the vdW gap tunneling spectroscopy, we fabricated another ~10 nm-thick MoS_2_ device (MS2; inset of Fig. 5d). The overall behaviors of the electrical properties of the MS_2_ device, such as the *n*-type behavior and the robust zero-conductance region (region ii) in Fig. 5d,e, respectively, show similar trends as those of the MS1 device. The conductance shoulders in Fig. 5f with the two vertical dashed lines provide an energy scale of ~ 0.92 eV at *V*_g_ = 64 V in region ii, which is also similar to that of the MS1 device.

**Figure 6:**
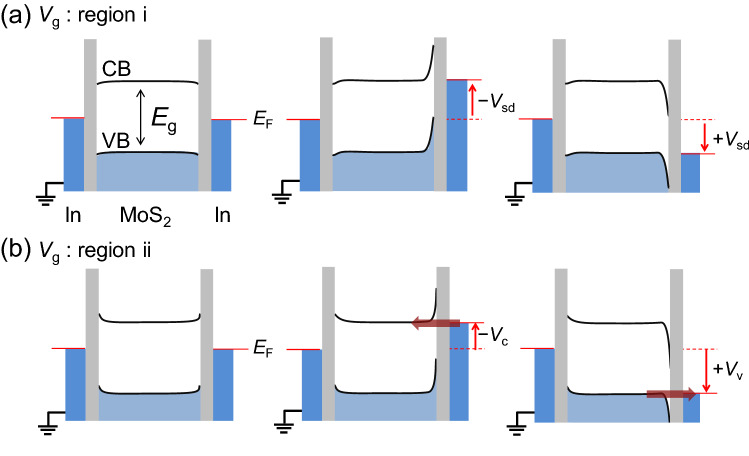
Band diagrams in MoS_2_ device with various *V*_g_ and *V*_sd_ values. (**a**), (**b**) Band diagrams with zero and finite *V*_sd_ conditions in *V*_g_ regions corresponding to regions “i” and “ii” depicted in Fig. 5b,e, respectively. *E*_F_ denotes the Fermi energy of In electrodes. Dark- and light-blue regions indicate bands filled by electrons of In and MoS_2_, respectively. The upper and lower solid lines in the MoS_2_ region represent the edges of the conduction and valence bands, respectively. *E*_g_ is the energy gap of MoS_2_. Solid red lines in the In region indicate Fermi levels depending on *V*_sd_ conditions. Vertical red arrows depict the direction of the changing Fermi levels with finite *V*_sd_ conditions from the zero-bias condition (dashed red lines). Horizontal red arrows represent the current flow directions via tunneling.

To determine the origin of the robust zero-conductance region, we considered the possibility of tunnel barriers with a high contact resistance due to the vdW gap between the MoS_2_ and In electrodes. Figure 6a,b show the band structures of MoS_2_ and In with vdW gap tunnel barriers at the MoS_2_/In interfaces (vertical gray bars) for representative *V*_g_ regions labeled as i and ii in Fig. 5b, respectively. For simplicity, we only considered the MS1 device in Fig. 5a–c. The upper and lower solid black curves correspond to the conduction-band (CB) and valence-band (VB) edges of MoS_2_, respectively. The light-blue region under the VB edge indicates the states occupied by electrons. For *V*_g_ ~  − 20 V, the MoS_2_ band was found to be shifted upward, whereas the band edges were fixed at the junction interfaces, where the left electrode was grounded. *E*_F(In)_ was located within the bandgap (*E*_g_) without *V*_sd_, as shown in the left panel of Fig. 6a (horizontal red line); thus, a sufficiently high *V*_sd_ is needed to overcome the *E*_g_ region. The middle and right panels of Fig. 6a show *V*_sd_ conditions in which the *E*_F(In)_ of the right electrode reaches the CB and VB edges, respectively. Nevertheless, electrons do not flow to the edges of the MoS_2_ because they experience a large Schottky barrier width. *V*_sd_ values greater than those corresponding to the band edges are thus needed to make a narrower Schottky barrier for the flow of electrons. With increasing *V*_g_, the bands for the MoS_2_ bend downward, leading to a relatively narrow Schottky barrier. Thus, the ± *V*_sd_ that allow the current to flow decrease with increasing *V*_g_, corresponding to region i in Fig. 5b. In region ii in Fig. 5b, the interval between ± *V*_sd_ locations for the finite conductance edges were found to change only slightly as *V*_g_ was varied. The band diagrams corresponding to region ii are plotted in Fig. 6b. In this region, the MoS_2_ electronic bands were observed to be substantially bent downward for *V*_g_ ~ 40 V. Here, the alignment of *E*_F(In)_ with the two band edges with proper *V*_sd_ values of − *V*_c_ and + *V*_v_ enabled the electrons to tunnel between the electrode and MoS_2_ under the assumption that the Schottky barrier widths were sufficiently narrow to allow the tunnel event, as indicated by horizontal arrows in the middle and right panels in Fig. 6b. The alignment resulted in no variation of the interval between − *V*_c_ and + *V*_v_ values, as indicated by the two dashed parallel lines in Fig. 5b. We note that the electrostatic band bending also occurs because of the vdW tunnel junction with a finite *V*_sd_, which leads to an additional band bending in a lower direction with the positive *V*_sd_.

The observed tunnel behavior with high contact resistances at In-contacted devices with 10 nm-thick (*n* ~ 15, where *n* is the number of MoS_2_ layers) MoS_2_ layers appears to be inconsistent with the behavior of In/MoS_2_ devices with MoS_2_ thicknesses ≤ 4 nm (*n* ≤ 6), which exhibit Ohmic behavior^13^. For this reason, we considered the location of *E*_F(In)_. Figure 5c,f show that the location of zero bias is nearly midway between the two vertical dashed lines, which implies that *E*_F(In)_ for *n* ~ 15 MoS_2_ is located near the midgap, indicating non-Ohmic contact. However, in the case of few-layer MoS_2_, *E*_F(In)_ is located just below the CB edge, resulting in Ohmic contact under the tunneling (or field-emission) mechanism through the vdW gap^13^. In this sense, performing vdW gap tunneling spectroscopy for *n* ≤ 6 is not possible, although the bandgap drastically increases from ~ 1.4 to ~ 1.9 eV when *n* is changed from 6 to 1^2^. Additional experimental and theoretical studies are needed to understand the current flowing between In and various-thickness MoS_2_ to know the limit of In-contacted vdW gap tunneling spectroscopy for MoS_2_.

## Conclusions

We carried out In-contacted vdW gap tunneling spectroscopy for 1*T*-TaS_2_, Bi-2212, and 2*H*-MoS_2_ using an FET geometry. We clearly observed the Mott gap (~ 0.4 eV), superconducting gap (~ 58 meV), and semiconducting bandgap (~ 0.9 eV) of 1*T*-TaS_2_, Bi-2212, and MoS_2_, respectively, by analyzing conductance curves as a function of *V*_sd_ at *T* = 4 K. Thus, we propose that vdW gap tunneling spectroscopy provides a feasible method to reveal the electronic band structure of inert vdW layered 2D materials. For semiconductor vdW materials of MoS_2_, we found that In-contacted vdW gap tunneling spectroscopy is applicable for only bulk MoS_2_ (*n* ~ 15), which could be related to the location of the Fermi level of In with respect to the midgap of MoS_2_. This reflects that the relative location of the Fermi level of In with respect to the midgap of vdW material may reveal the limitations of In-contacted vdW gap tunneling spectroscopy. For non-semiconductor vdW materials of 1*T*-TaS2 and Bi-2212 with thickness of tens of nanometers, we confirmed the In-contacted vdW gap tunneling spectroscopy is applicable while we need further study for the validity for a few layers.

## Methods

### Samples

We fabricated electrical devices with In contacts for TaS_2_, Bi-2212, and MoS_2_ on 500 nm-thick SiO_2_/Si substrates by conventional microfabrication processes. We deposited 100 nm-thick In electrodes onto a multilayer flake in a thermal evaporator at a vacuum of 3 × 10^−6^ Torr. The substrate was attached to a stage, which was cooled to 100 K using liquid N_2_ during the In deposition process. The low temperature of the sample stage ensured a homogeneously deposited In layer with a uniform thickness and without grain boundaries^16^.

### Measurements

We performed electrical measurements using a two-probe configuration in a closed-cycle refrigerator with a base temperature of 4 K. Bias voltages were applied by Keithley 213 quad voltage source and current was measured by a current amplifier (Ithaco 1211, DL).

### Computations

DFT calculations for Fig. 1b,c are carried out within generalized gradient approximation (GGA) for exchange–correlation (*xc*) functionals^30,31^, implemented in the Vienna ab initio simulation package (VASP)^32^.

## Supplementary Information

Supplementary Information.

## References

[1] Sipos B (2008). From Mott state to superconductivity in 1*T*-TaS_2_. Nat. Mater..

[2] Yu Y (2015). Gate-tunable phase transitions in thin flakes of 1*T*-TaS_2_. Nat. Nanotechnol..

[3] Costanzo D, Jo S, Berger H, Morpurgo AF (2016). Gate-induced superconductivity in atomically thin MoS_2_ crystals. Nat. Nanotechnol..

[4] Saito Y (2016). Superconductivity protected by spin–valley locking in ion-gated MoS_2_. Nat. Phys..

[5] Ugeda MM (2016). Characterization of collective ground states in single-layer NbSe_2_. Nat. Phys..

[6] Chen X (2015). Probing the electron states and metal-insulator transition mechanisms in molybdenum disulphide vertical heterostructures. Nat. Commun..

[7] Xi X (2015). Strongly enhanced charge-density-wave order in monolayer NbSe_2_. Nat. Nanotechnol..

[8] Jiang D (2014). High-Tc superconductivity in ultrathin Bi_2_Sr_2_CaCu_2_O_8+x_ down to half-unit-cell thickness by protection with graphene. Nat. Commun..

[9] Yu Y (2019). High-temperature superconductivity in monolayer Bi_2_Sr_2_CaCu_2_O_8+δ_. Nature.

[10] Zareapour P (2012). Proximity-induced high-temperature superconductivity in the topological insulators Bi_2_Se_3_ and Bi_2_Te_3_. Nat. Commun..

[11] Li AJ, Zhu X, Stewart GR, Hebard AF (2017). Bi-2212/1T-TaS_2_ Van der Waals junctions: Interplay of proximity induced high-*T*_c_ superconductivity and CDW order. Sci. Rep..

[12] Liao M (2018). Superconductor-insulator transitions in exfoliated Bi_2_Sr_2_CaCu_2_O_8+x_ flakes. Nano Lett..

[13] Cui X (2015). Multi-terminal transport measurements of MoS_2_ using a van der Waals heterostructure device platform. Nat. Nanotechnol..

[14] Wang Y (2019). Van der Waals contacts between three-dimensional metals and two-dimensional semiconductors. Nature.

[15] Kim B-K (2021). Origins of genuine Ohmic van der Waals contact between indium and MoS_2_. npj 2D Mater. Appl..

[16] Choi D-H (2017). Van-der-Waals-gap tunneling spectroscopy for single-wall carbon nanotubes. Carbon.

[17] Choi D-H (2021). Tunneling spectroscopy for electronic bands in multi-walled carbon nanotubes with van der waals gap. Molecules.

[18] Kim J-J, Yamaguchi W, Hasegawa T, Kitazawa K (1994). Observation of Mott localization gap using low temperature scanning tunneling spectroscopy in commensurate 1T-TaS_2_. Phys. Rev. Lett..

[19] Dardel B (1992). Temperature-dependent pseudogap and electron localization in 1T-TaS_2_. Phys. Rev. B.

[20] Kim J-J, Ekvall I, Olin H (1996). Temperature-dependent scanning tunneling spectroscopy of 1T-TaS_2_. Phys. Rev. B.

[21] Cho D (2016). Nanoscale manipulation of the Mott insulating state coupled to charge order in 1*T*-TaS2. Nat. Commun..

[22] Inada R, Ōnuki Y, Tanuma S (1980). Hall effect of 1T-TaS_2_ and 1T-TaSe_2_. Physica B+C.

[23] Konstantinović Z, Li ZZ, Raffy H (2000). Temperature dependence of the Hall effect in single-layer and bilayer bilayer Bi_2_Sr_2_Ca_n__−__1_Cu_n_O_y_ thin films at various oxygen contents. Phys. Rev. B.

[24] Sterpetti E, Biscaras J, Erb A, Shukla A (2017). Comprehensive phase diagram of two-dimensional space charge doped Bi_2_Sr_2_CaCu_2_O_8+x_. Nat. Commun..

[25] Bae M-H, Park J-H, Choi J-H, Lee H-J, Park K-S (2008). Pseudogap behavior revealed in interlayer tunneling in overdoped Bi_2_Sr_2_CaCu_2_O_8+x_. Phys. Rev. B.

[26] Kordyuk AA (2015). Pseudogap from ARPES experiment: Three gaps in cuprates and topological superconductivity. Low Temp. Phys..

[27] Hashimoto M, Vishik IM, He R-H, Devereaux TP, Shen Z-X (2014). Energy gaps in high-transition-temperature cuprate superconductors. Nat. Phys..

[28] Prozorov R, Giannetta RW (2006). Magnetic penetration depth in unconventional superconductors. Supercond. Sci. Technol..

[29] Mak KF, Lee C, Hone J, Shan J, Heinz TF (2010). Atomically thin MoS_2_: A new direct-gap semiconductor. Phys. Rev. Lett..

[30] Kohn W, Sham LJ (1965). Self-consistent equations including exchange and correlation effects. Phys. Rev..

[31] Perdew JP, Burke K, Ernzerhof M (1996). Generalized gradient approximation made simple. Phys. Rev. Lett..

[32] Kress G, Furthmüller J (1996). Efficient iterative schemes for ab initio total-energy calculations using a plane-wave basis set. Phys. Rev. B.

