# Supplementary material for: Indium-contacted van der Waals gap tunneling spectroscopy for van der Waals layered materials
Source: Sci Rep. 2021 Sep 7;11:17790. doi: 10.1038/s41598-021-97110-z (PMC8423830; doi:10.1038/s41598-021-97110-z)
Supplement: Supplementary file 1 — Supplementary Information. [file 41598_2021_97110_MOESM1_ESM.docx]

Supplementary Information for

**Indium-contacted van der Waals gap tunneling spectroscopy for van der Waals layered materials**

Dong-Hwan Choi^1,2^, Kyung-Ah Min^3^, Suklyun Hong^3^, Bum-Kyu Kim^2^, Myung-Ho Bae^2,4*^ and Ju-Jin Kim^1*^

^1^Department of Physics, Jeonbuk National University, Jeonju 54896, Republic of Korea

^2^Korea Research Institute of Standards and Science, Daejeon 34113, Republic of Korea

^3^Department of Physics and Graphene Research Institute, Sejong University, Seoul 05006, Korea

^4^Department of Nano Science, University of Science and Technology, Daejeon, 34113, Republic of Korea

^*^E-mail: mhbae@kriss.re.kr, jujinkim@chonbuk.ac.kr

Supplementary Figure 1. Resistivity (*ρ*) as a function of temperature (*T*) of 1*T*-TaS_2_ in a four-probe measurement (contacted metal: Ti/Au). Arrows indicate the temperature sweep directions.

Supplementary Figure 2. (a),(b) Hall mobility (*μ*_H_) and carrier density (*n*_H_) of 6 nm thick MoS_2_ on a hexagonal-BN substrate as a function of temperature (*T*) for various back-gate voltage (*V*_g_) conditions. Inset of (a): optical image of MoS_2_ device with 85 nm thick indium (In) contacts. Scale bar: 2 μm.
